# Characteristics of the nuclear (18S, 5.8S, 28S and 5S) and mitochondrial (12S and 16S) rRNA genes of *Apis mellifera* (Insecta: Hymenoptera): structure, organization, and retrotransposable elements

**DOI:** 10.1111/j.1365-2583.2006.00689.x

**Published:** 2006-10-01

**Authors:** J J Gillespie, J S Johnston, J J Cannone, R R Gutell

**Affiliations:** * Department of Entomology, Texas A & M University College Station, TX, USA; † Department of Microbiology and Immunology, University of Maryland School of Medicine Baltimore, MD, USA; ‡ Virginia Bioinformatics Institute at Virginia Tech Blacksburg, VA, USA; § Institute for Cellular and Molecular Biology and Section of Integrative Biology, University of Texas Austin, TX, USA

**Keywords:** honey bee, *Apis mellifera*, ribosomal RNA, rRNA, rDNA, 18S, 5.8S, 28S, 5S, 16S, 12S, ITS-1, ITS-2, ETS, IGS, secondary structure, alignment, retrotransposition, R1, R2, R element, reverse transcriptase

## Abstract

As an accompanying manuscript to the release of the honey bee genome, we report the entire sequence of the nuclear (18S, 5.8S, 28S and 5S) and mitochondrial (12S and 16S) ribosomal RNA (rRNA)-encoding gene sequences (rDNA) and related internally and externally transcribed spacer regions of *Apis mellifera* (Insecta: Hymenoptera: Apocrita). Additionally, we predict secondary structures for the mature rRNA molecules based on comparative sequence analyses with other arthropod taxa and reference to recently published crystal structures of the ribosome. In general, the structures of honey bee rRNAs are in agreement with previously predicted rRNA models from other arthropods in core regions of the rRNA, with little additional expansion in non-conserved regions. Our multiple sequence alignments are made available on several public databases and provide a preliminary establishment of a global structural model of all rRNAs from the insects. Additionally, we provide conserved stretches of sequences flanking the rDNA cistrons that comprise the externally transcribed spacer regions (ETS) and part of the intergenic spacer region (IGS), including several repetitive motifs. Finally, we report the occurrence of retrotransposition in the nuclear large subunit rDNA, as R2 elements are present in the usual insertion points found in other arthropods. Interestingly, functional R1 elements usually present in the genomes of insects were not detected in the honey bee rRNA genes. The reverse transcriptase products of the R2 elements are deduced from their putative open reading frames and structurally aligned with those from another hymenopteran insect, the jewel wasp *Nasonia* (Pteromalidae). Stretches of conserved amino acids shared between *Apis* and *Nasonia* are illustrated and serve as potential sites for primer design, as target amplicons within these R2 elements may serve as novel phylogenetic markers for Hymenoptera. Given the impending completion of the sequencing of the *Nasonia* genome, we expect our report eventually to shed light on the evolution of the hymenopteran genome within higher insects, particularly regarding the relative maintenance of conserved rDNA genes, related variable spacer regions and retrotransposable elements.

## Introduction

Ribosomal RNA (rRNA)-encoding genes (rDNA) and related genetic elements have been well studied for over six decades (see Noller, 2005 for a recent review), with interests ranging from pharmaceutical and biochemical investigations to comparative biological studies garnering a wealth of information on the structural, functional and evolutionary characteristics of these molecules. Phylogenetic studies, in particular, have propagated a large number of rRNA gene sequences on public genetic databases, as the organismal universality and typically high gene copy number per cell facilitate gene amplification and sequencing (e.g. Woese, 1987; Woese *et al*., 1990; Winker & Woese, 1991). Within rRNA genes, varying evolutionary rates of base substitution can be localized to conserved core rRNA sequences (e.g. Gutell *et al*., 1985; Van de Peer *et al*., 1993; Vawter & Brown, 1993; Sullivan *et al*., 1995; Cannone *et al*., 2002; Misof *et al*., 2002; Baele *et al*., 2006) as well as rapidly evolving variable regions and expansion segments (e.g. Hassouna *et al*., 1984; Gerbi, 1985, 1996; Gorski *et al*., 1987; Neefs & De Wachter, 1990; Hancock, 1995; Schnare *et al*., 1996). Rates of substitution are even faster in transcribed spacer regions and non-coding spacer regions, which can reveal high levels of divergence even when compared across closely related species (e.g. Long & Dawid, 1980; Hillis & Dixon, 1991; Osorio *et al*., 2005), as well as within individual genomes (e.g. Gruendler *et al*., 1991; Lanadu *et al*., 1992; Lakshmikumaran & Negi, 1994; Rocheford, 1994). These conserved and variable regions of rRNA genes have not only been useful for recovering phylogenetic relationships (e.g. Woese *et al*., 1990; Friedrich & Tautz, 1997a,b), but also for understanding the structural and functional constraints of both the coding and the non-coding organizational sequences within rRNA genes (see Noller, 2005).

Recent crystal structures of the ribosome (Cate *et al*., 1999; Ban *et al*., 2000; Schluenzen *et al*., 2000; Wimberly *et al*., 2000; Spahn *et al*., 2001; Yusupov *et al*., 2001) have verified many of the long-standing structures of the rRNAs that were predicted by comparative sequence analysis (Woese *et al*., 1980; Noller & Woese, 1981; Noller *et al*., 1981; Brimacombe *et al*., 1983; Gutell *et al*., 1985, 1986; Woese & Pace, 1993) or substantiated with site-directed or random mutagenesis (e.g. Musters *et al*., 1989, 1991; Sweeney & Yao, 1989; Green *et al*., 1990). Still, these three-dimensional models have yielded further information on the organization of the small and large subunits (SSU and LSU, respectively) with the numerous ribosomal proteins comprising the mature ribosome. In particular, new information on enzymatic function (e.g. Nissen *et al*., 2000), non-canonical base pairing (e.g. Leontis & Westhof, 2003; Lee & Gutell, 2004; Lescoute *et al*., 2005), tertiary interactions via coaxial stacking (e.g. Elgavish *et al*., 2001; Ogle *et al*., 2001), RNA turn motifs (e.g. Gutell *et al*., 2000; Klein *et al*., 2001), rRNA–protein interactions (e.g. Brodersen *et al*., 2002; Hoang *et al*., 2004; Klein *et al*., 2004a; Noller, 2004), metal ions and rRNA–rRNA packing interactions (Klein *et al*., 2004b), lone pair triloops (Lee *et al*., 2003) and dynamism (Carter *et al*., 2001; Harms *et al*., 2001; Noller & Baucom, 2001) has been elicited from observations of tertiary models not easily obtained from comparative sequence analysis. It is essential that these characteristics be incorporated into multiple sequence alignments to improve the assignment of structurally and functionally homologous bases, as well as to verify that generated sequences are indeed functional rRNA genes and not paralogues and/or pseudogenes (e.g. Buckler *et al*., 1997; Muir *et al*., 2001; Bailey *et al*., 2003; Márquez *et al*., 2003) or artefacts of the sequencing process (see Clark & Whittam, 1992; States, 1992; Hickson *et al*., 1996; Gillespie *et al*., 2005a).

In this study, we predict entire secondary and some tertiary structures of the nuclear (nl) and mitochondrial (mt) SSU and LSU rRNAs from the honey bee, *Apis mellifera*, as the recent compilation of its genome (The Honey Bee Genome Sequencing Consortium, 2006) has provided sequences spanning the entire rDNA region. Wherever possible, we include information from published crystal structures of the ribosome and chemical probing studies to annotate and update diagrams of arthropod nl and mt rRNA structure. These diagrams (and related multiple sequence alignments) will be valuable for related studies on the phylogeny of arthropods, as well as the evolution of the structure and function of eukaryotic and organellar rRNAs.

With the completion of the honey bee genome comes the opportunity to begin analysing the intragenomic variation associated with repetitive sequence motifs, particularly those associated with multicopy genes. Here, we make an attempt to characterize the sequences associated with the externally transcribed spacer regions (ETS) and intergenic spacers (IGS) that are conserved across all rDNA cistrons in the honey bee genome. We describe several repeat regions that occur in the 5′- and 3′-ends of the IGS regions. Our preliminary mapping of these complete ETS sequences and partial IGS sequences will be useful for future studies that attempt to span the further repetitive motifs of the honey bee rRNA genes that occur in the central portion of the IGS, including promoter and enhancer regions.

As a result of the compilation of all rRNA genes in the honey bee genome, we were able to assess the degree of rRNA gene inactivation by the insertion of retrotransposable elements. Two types of retrotransposable elements, R1 and R2, can disrupt the complete transcription of arthropod rRNA genes by inserting into conserved sequences of the nl LSU rDNA (e.g. Long & Dawid, 1979; Jamrich & Miller, 1984; Jakubczak *et al*., 1990, 1991; Burke *et al*., 1993, 1999; Eickbush, 2002). Our analysis of R1 and R2 element retrotransposition in the honey bee rRNA genes is somewhat consistent with other studies on arthropod retrotransposition, and we provide here the predicted open reading frame (ORF) of an R2 element retrotransposase (RT).

## Results and discussion

### Predicted rRNA secondary structures

**Figure 1:**
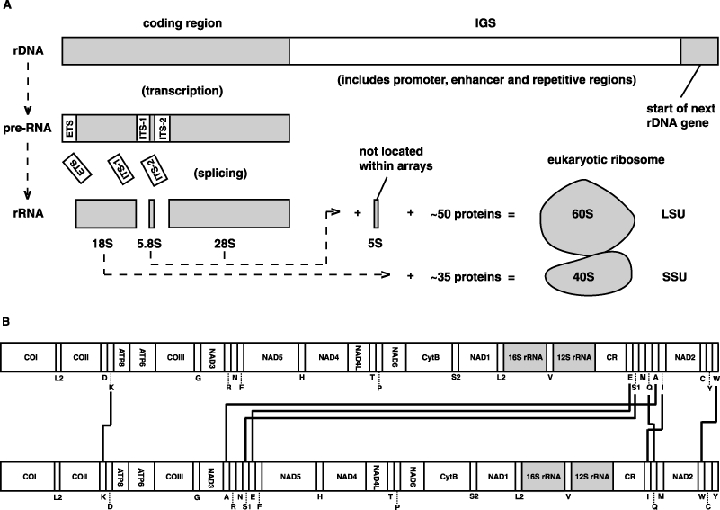
Organization of the rRNA genes within the honey bee genome. (A) Typical organization of the nuclear rRNA genes of eukaryotes. IGS, intergenic spacer; SSU, small subunit; LSU, large subunit. (B) Position of the rRNA genes (shaded) within the honey bee mitochondrial genome (top) and the ancestral arthropod mitochondrial genome (bottom). Rearrangements in the honey bee genome are depicted with solid lines. See Clary & Wolstenholme (1985, 1987) and Crozier & Crozier (1993) for information on honey bee mitochondrial genes.

Our results are presented within the typical organization and pattern of transcription of eukaryotic rRNA genes. A schematic diagram illustrates the composition of typical arthropod rRNA genes within the nuclear and mitochondrial genomes (Fig. 1). Within the honey bee genome, the nl rRNA gene arrays have been identified as occurring on two different chromosomes (Beye & Moritz, 1993) subsequently localized to linkage groups 6 and 12 (Aquino-Perez, unpublished data), while the mt rRNA genes are organized in the ancestral arthropod position (see Boore *et al*., 1995; Boore, 1999) within the mitochondrial genome (Crozier & Crozier, 1993). In insects, pioneering structural studies of rRNA molecules were conducted on flies (Diptera) (Clary & Wolstenholme, 1985, 1987; Gutell & Fox, 1988; Hancock & Dover, 1988; Hancock *et al*., 1988; Tautz *et al*., 1988) and one beetle (Coleoptera) (Hendriks *et al*., 1988a). While these studies predicted structures mostly from comparisons with other early published complete rDNA sequences from bacteria (Glotz & Brimacombe, 1980; Woese *et al*., 1980; Noller & Woese, 1981; Noller *et al*., 1981; Stiegler *et al*., 1981; Zwieb *et al*., 1981), yeast (Veldman *et al*., 1981), frog (Ware *et al*., 1983; Clark *et al*., 1984; Dunon-Bluteau & Brun, 1986), rodent (Bibb *et al*., 1981; Hassouna *et al*., 1984; Michot *et al*., 1984; Gutell *et al*., 1985), cow (Gutell *et al*., 1985), human (Gorski *et al*., 1987) and other mammals (Hixson & Brown, 1986; Wool, 1986), they were lacking a larger collection of arthropod sequences to make thorough comparisons of most of the variable regions outside of the core rRNA [although see the early structural model of the mt LSU rRNA of the bird spider (Hendriks *et al*., 1988b)]. Thus, the following sections review subsequent structural studies that were performed with comparisons across several to many arthropod/insect rDNA sequences.

#### Nuclear SSU rRNA

**Figure 2:**
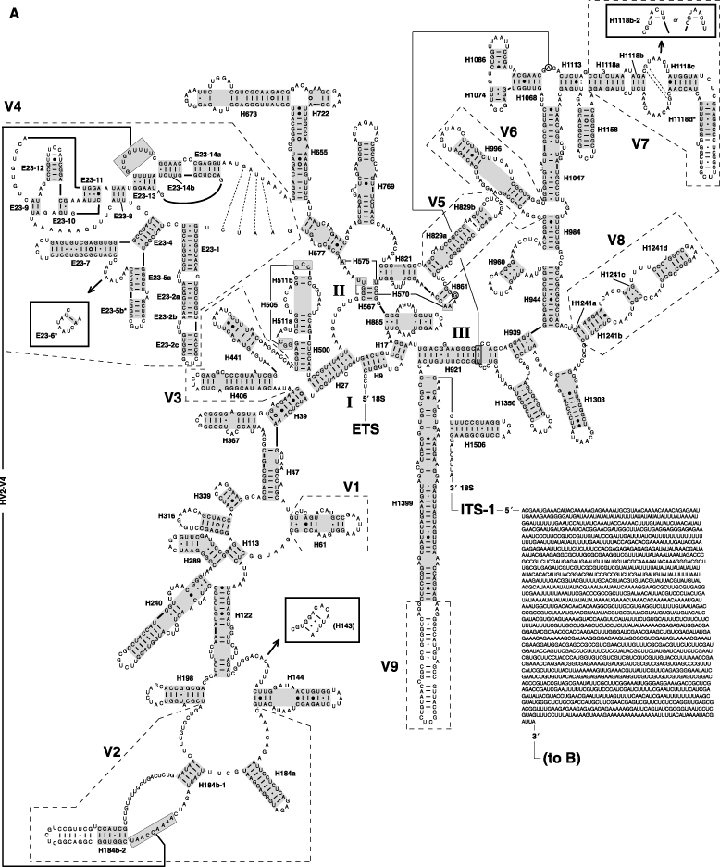

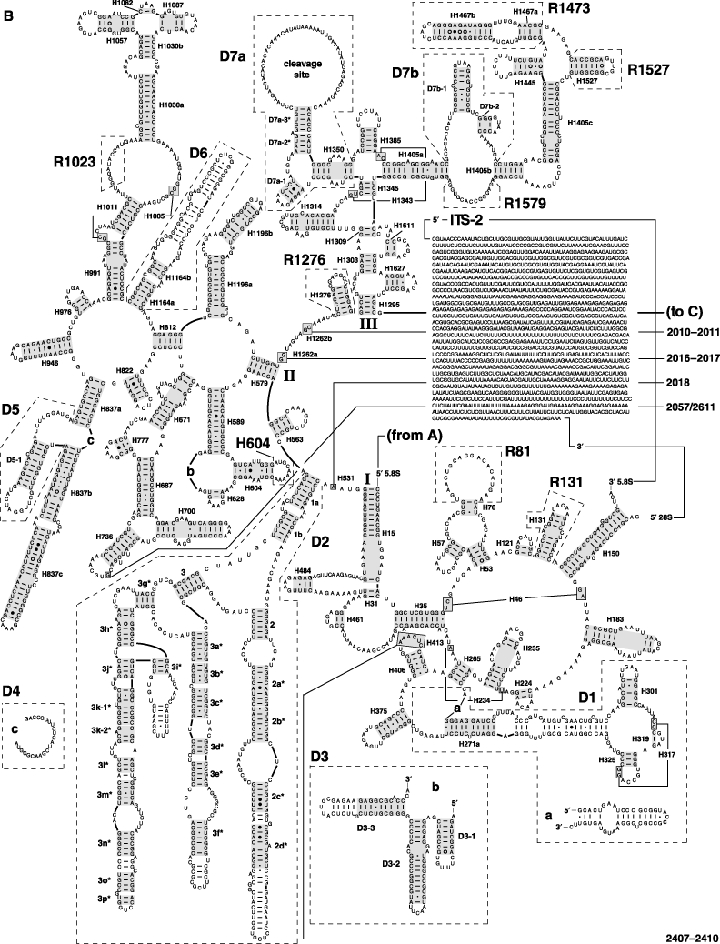

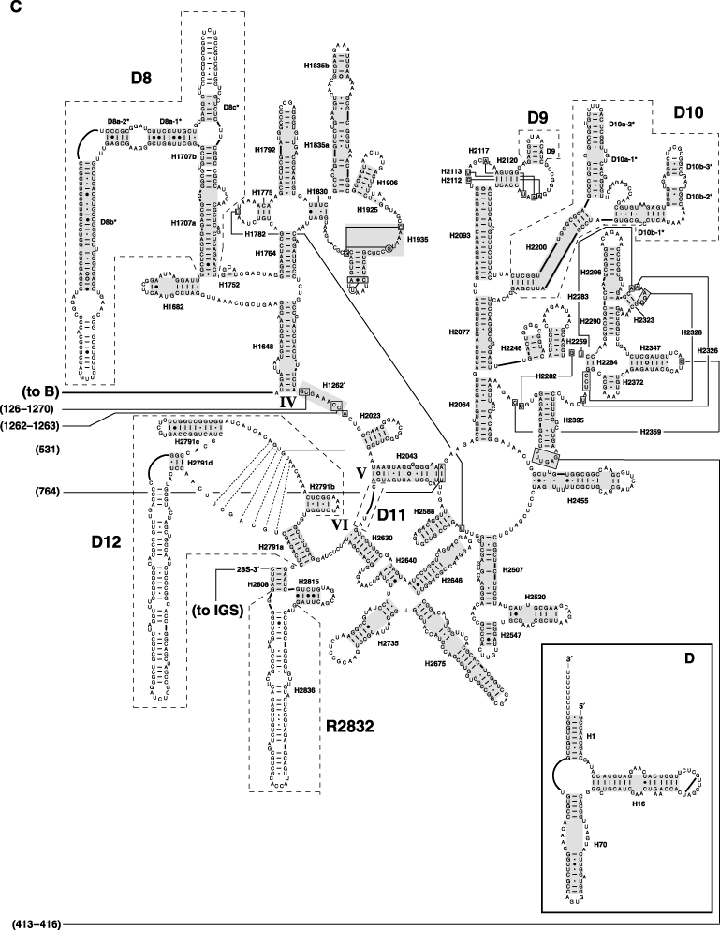
The secondary structure model of the nuclear rRNA (18S + 5.8S + 28S + 5S) from the honey bee, *Apis mellifera*. Variable regions are enclosed within dashed boxes and the naming follows either Schnare *et al*. (1996), Van de Peer *et al*. (1999) or Gillespie *et al*. (2005a,b,c). Helix numbering follows the system of Cannone *et al*. (2002), except for variable region 4 (V4) for which the notation of Wuyts *et al*. (2000) and Gillespie *et al*. (2005a) is used. Helices aligned across all sampled panarthropods are boxed in grey. Tertiary interactions (where there is strong comparative support) and base triples are shown connected by continuous lines. Base pairing is indicated as follows: standard canonical pairs by lines (C-G, G-C, A-U, U-A); wobble G·U pairs by dots (G·U); A·G pairs by open circles (A○G); other non-canonical pairs by filled circles (e.g. C•A). (A) Domains I–III of SSU rRNA (18S). Regions with alternative structures are boxed. (B) Domains I–III of LSU rRNA (5.8S + 28S). (C) Domains IV-VI of LSU rRNA (28S). (D) 5S rRNA. Diagrams were generated using the program XRNA (Weiser, B. & Noller, H., University of California at Santa Cruz) with manual adjustment.

This model (Fig. 2A) is in concordance with the conserved 16S (prokaryote) and 16S-like (eukaryote) structures of the SSU rRNA from the literature (Woese *et al*., 1980; Noller & Woese, 1981; Stiegler *et al*., 1981; Gutell *et al*., 1985; Huysmans & De Wachter, 1986; Dams *et al*., 1988; Neefs *et al*., 1990, 1991, 1993; De Rijk *et al*., 1992; Gutell, 1994, 1996; Van de Peer *et al*., 1994, 1996, 1997, 1998, 1999; De Rijk *et al*., 1998; Wuyts *et al*., 2004) and is based on an updated model of *Drosophila melanogaster* (Gutell, 1993, 1994; Van de Peer *et al*., 2000; Cannone *et al*., 2002). Aside from *D. melanogaster*, structural models of five insect taxa have been published, including *Tenebrio molitor* (Coleoptera) (Hendriks *et al*., 1988a), *Acyrthosiphon pisum* (Heteroptera) (Kwon *et al.*, 1991), peloridiid Hemiptera (Ouvrard *et al.*, 2000), *Loricera foveata* (Coleoptera) (Van de Peer *et al.*, 2000) and ichneumonoid Hymenoptera (Gillespie *et al.*, 2005c). Our model is based loosely on these earlier models, and relies more heavily on the structure predictions from three recent studies wherein the 18S rRNA secondary structure was predicted across most insect orders (Kjer, 2004; Gillespie *et al.*, 2005a; Yoshizawa & Johnson, 2005) as well as evidence from SSU rRNA crystal structures (Schluenzen *et al.*, 2000; Wimberly *et al.*, 2000; Yusupov *et al.*, 2001). For referencing purposes, our new model follows the convention (variable region nomenclature, helix numbering, etc.) put forth by Gillespie *et al.* (2005a). The SSU rRNA contains 65 helices that are conserved across arthropods (Fig. 2A).

The predicted structure of most regions of the 18S rRNA from honey bee are consistent with structures of the major lineages of Arthropoda, and differences between the nl SSU rRNA model of Gillespie *et al*. (2005a) and that presented here are illustrated on the jRNA website (see Experimental procedures). Variable region four (V4) (Neefs & De Wachter, 1990; Van de Peer *et al*., 1999) is shown with refinements to the double pseudoknot model of Wuyts *et al*. (2000), such that their proposed base triple is removed in place of the recently predicted tertiary interaction between the hairpin loop of the second pseudoknot and an unpaired sequence in variable region 2 (V2) (Alkemar & Nygård, 2003, 2004). Our alignment across arthropod V4 sequences spanning most orders shows strong support for the internal seven base pairs of this helix (**HV2–V4**); however, base pairs 1 and 9 are minimally supported with covariation evidence across arthropods (CRW Site bp frequency tables; Gillespie *et al*., 2005a).

**Figure 3:**
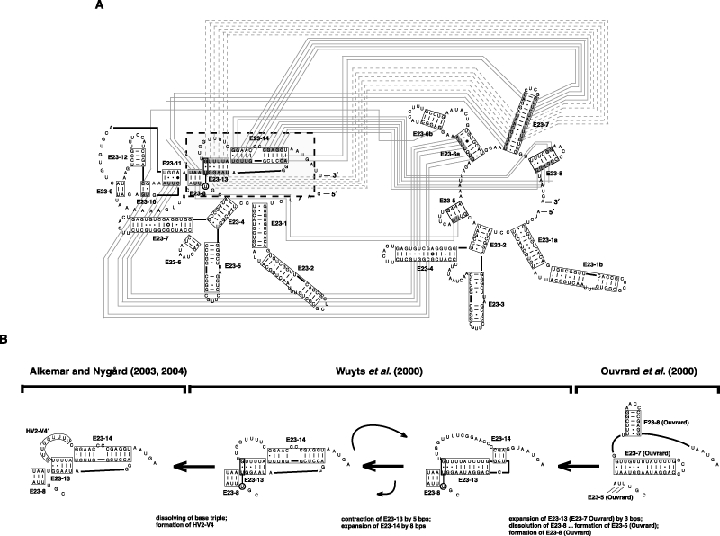
An illustration of several proposed secondary structural models for variable region V4 (V4) of arthropod 18S rRNA. (A) A comparison of the Wuyts *et al*. (2000) model (left) with the Ouvrard *et al*. (2000) model. Nucleotides in the 3′-half of V4 that are base paired in both models are shaded. Shaded nucleotides forming different base pairs in each model are connected with dashed lines. Shaded nucleotides forming the same base pairs in both models are connected with solid lines. The region within the dashed box is illustrated in (B). (B) An example of a possible dynamic relationship between three proposed models for the 18S○V4. See Fig. 2 legend for explanations of base pair symbols, helix numbering and reference for software used to construct structure diagrams.

Accurate structural models of arthropod V4 are of particular interest because this region of 18S rRNA is the most commonly sequenced marker for higher level arthropod phylogeny estimation (reviewed in Kjer, 2004). Still, multiple structural predictions exist for this large variable region of SSU rRNA. For instance, Ouvrard *et al*. (2000) proposed a secondary structure model for insect V4 based on the comparative analysis of 22 insect sequences. That same year, Wuyts *et al*. (2000) presented a refined model with the 3′-half of this variable region consisting of two pseudoknots. One of the pseudoknots was previously predicted (Neefs & De Wachter, 1990), while the other, a rare type of pseudoknot similar to that found in plant viruses (van Batenburg *et al*., 2000), was newly reported. Within these two contrasting models of the 3′-half of arthropod V4, 32 nucleotides form different base pairs (Fig. 3A). Six nucleotides form the same base pairs but within different structural contexts (grey dashed lines in Fig. 3A). Interestingly, across most eukaryotes this region alternates the number of base pairs within helices **E23-13** and **E23-14** (Wuyts *et al*., 2000). Our analysis of honey bee (and most arthropod) 18S rRNA sequences is consistent with an expanded helix **E23-13** (6–11 bp) and a contracted helix **E23-14** (11–3 bp) (Fig. 3B). A base pair frequency table (available at the jRNA website) suggests that the model of Wuyts *et al*. (2000), including HV2–V4 (Alkemar & Nygård, 2003, 2004), is more probable based on more covariation at the positions in the proposed base pairs; however, a minimal amount of compensatory base change evidence supports the Ouvrard *et al*. (2000) model such that a dynamic nature of this region of nl SSU rRNA cannot be disproved. These findings are consistent with a recent comparison of the Wuyts *et al*. (2000) and Alkemar & Nygård (2003, 2004) models in the V2 region across most insect lineages (Gillespie *et al*., 2005a; see table 3).

Of the remaining regions of insect 18S rRNA, domain I (including variable regions V1–V3) is sequenced less frequently than domain III (including variable regions V6–V9) (Cannone *et al*., 2002). Variable regions V1, V3, V6 and V8 (and V5 of domain II) are considerably smaller in size than V4 and yield little phylogenetic information (Gillespie *et al*., 2005a). The V2 region is difficult to align with confidence due to length variation. Studies on arthropod V7 have divulged a region characteristic of extraordinary variation in the length of helices (Crease & Colbourne, 1998; Crease & Taylor, 1998; McTaggart & Crease, 2005). Sequence alignments of V7 across divergent insect taxa contain many variable positions, yet structure is necessary to guide the alignment. This is because, while only one helix is present in nearly all non-holometabolous orders, as well as in Amphiesmenoptera (Lepidoptera + Trichoptera) (Gillespie *et al*., 2005a), the remaining seven (minus Thysanoptera) holometabolous orders contain a second helix within V7. Additionally, this smaller less-conserved helix, **H1118b-2** (Gillespie *et al*., 2005a), can be present or absent within these orders, with its base pairs often containing minimal comparative support. Finally, sometimes it is difficult to predict base pairing in the V9 at the end of the penultimate helix because many of the published sequences are missing the extreme 3′-end of the 18S rRNA (Cannone *et al*., 2002).

#### Nuclear LSU rRNA

This model is in concordance with the conserved 23S (prokaryote) and 23S-like (eukaryote) structures of the LSU rRNA from the literature (Noller *et al*., 1981; Wool, 1986; Gutell & Fox, 1988; Gutell *et al*., 1990, 1992, 1993; Larsen, 1992; De Rijk *et al*., 1994, 1997, 2000; Gutell, 1996; Schnare *et al*., 1996; Wuyts *et al*., 2001, 2002, 2004). With existing predicted structure models for *Aedes albopictus* (Kjer *et al*., 1994), *D. melanogaster* (Schnare *et al*., 1996; others therein), *Acyrthosiphon pisum* (Amako *et al*., 1996), *Tenebrio* sp. (Gillespie *et al*., 2004) and ichneumonoid Hymenoptera (Gillespie *et al*., 2005b), this is the sixth predicted structure of a complete or nearly complete insect nl LSU rRNA (Fig. 2B,C). To date, complete comparative models (alignments) for insect 28S rRNA have not been published, so we present our model following the nearly complete model of ichneumonoid Hymenoptera (Gillespie *et al*., 2005b) with adherence to LSU rRNA crystal structures (Cate *et al*., 1999; Ban *et al*., 2000; Spahn *et al*., 2001; Yusupov *et al*., 2001). Eukaryotic nl LSU rRNA is interrupted by an internally transcribed spacer region (ITS-2) in the terminal loop of helix **H150** that separates the LSU into 5.8S and 28S rRNAs (Veldman *et al*., 1981; Michot *et al*., 1982). Flies (Diptera) have a second ITS in the nl LSU that occurs within the terminal loop of helix **H131** (Tautz *et al*., 1988). The *A. mellifera* LSU rRNA (minus 5S rRNA) structure model contains 143 helices (133 in the 28S rRNA and five in the 5.8S rRNA, with five helices comprised of both 28S and 5.8S rRNAs) that are conserved across arthropods (Fig. 2B,C).

Thirteen regions previously described as extremely variable or expansion segments (Clark *et al*., 1984) of the 28S rRNA sequence are difficult to align across the arthropods (e.g. Hwang *et al*., 1998). These regions of honey bee rRNA have the typical variation seen across insect orders and families. Regarding Hymenoptera, the structures for two of the largest of these regions, expansion segments D2 and D3, were recently predicted in studies of 349 ichneumonoid wasps (Gillespie *et al*., 2005c; Wharton *et al*., 2006) and are comparable to the model predicted across 527 chalcidoid wasps (Gillespie *et al*., 2005b) and 60 evanioid wasps (Deans *et al*., 2006). Two other studies have used secondary structure as an alignment guide for phylogeny estimation of hymenopteran D2 sequences but did not produce global structural models (Belshaw & Quicke, 1997, 2002). While expansion segment D2 is phylogenetically informative within orders and families of arthropods (for a comparison of our model with Coleoptera see Gillespie *et al*., 2004), the alignment of these sequences across highly divergent taxa (i.e. class and phylum levels) is extraordinarily difficult (see Schnare *et al*., 1996). However, the expansion segment D3 is conserved enough in its secondary structure to be of practical use in arthropod phylogeny estimation (e.g. Whiting *et al*., 1997; Wheeler *et al*., 2001; Hovmöller *et al*., 2002), although its phylogenetic signal is weak when analysed without other data partitions (see Kjer, 2004).

As expansion segments D2 and D3 of the 28S rRNA can be amplified in one PCR reaction, and because they usually contain characteristic variation in sequence length and base composition that is informative across family, genus and species levels, this region of nl LSU rRNA has become one of the most common phylogenetic markers (e.g. Gillespie *et al*., 2003; Schulmeister, 2003). Thus, the majority of published insect nl LSU rRNA sequences are comprised of expansion segments D2 and D3 and related core sequences (Cannone *et al*., 2002). Some insect phylogenetic studies have generated sequences for expansion segments D4 to D10 (e.g. Hwang *et al*., 1998; Belshaw & Quicke, 2002; Wiegmann *et al*., 2003); however, in-depth structural predictions with reference to previous models were not provided. This lack of structural information from these studies, coupled with limited sequences covering the majority of the 3′-half of the 28S rRNA, makes it difficult to propose models that are thoroughly supported with base pair covariation. Therefore, our proposed structures for this region of the nl LSU rRNA, particularly expansion segments D7, D10, D12 and R2832, should not be considered well supported. Our labs are currently generating more sequences from the 3′-half of the 28S rRNA from a wide range of insect taxa that should improve these preliminary structure predications.

Sequences beyond the D3 in domain II include expansion segments D4–D6, which are relatively small for candidate phylogenetic markers (Fig. 2B). Earlier predicted structures for these regions in insects (Hancock *et al*., 1988; Kjer *et al*., 1994; Hwang *et al*., 1998) have recently been modified based on a more thorough taxon sampling (Gillespie *et al*., 2005c). In domain IV, a notable expansion segment, D7a, has been identified as the region of metazoan nl LSU rRNA wherein an internally processed cleavage site termed the ‘hidden break’ occurs. As originally characterized in the dipteran *Sciara coprophila* (Ware *et al*., 1985), this cleavage site separates the 28S rRNA into α and β fragments of roughly equal size (Applebaum *et al*., 1966; Balazas & Agosin, 1968; Greenberg, 1969; Ishikawa & Newburgh, 1972; Ishikawa, 1977; de Lanversin & Jacq, 1989). Aside from pea aphid (*Acyrthosiphon pisum*) 28S rRNA (Ogino *et al*., 1990); which does not separate into α and β fragments, most insects contain this hidden break (e.g. Shine & Dalgarno, 1973; Park & Fallon, 1990), which was speculated to occur near an unpaired 5′-UAAU-3′ sequence within expansion segment D7 (Ware *et al*., 1985). Despite this prevalent pattern, the proposed cleavage signal is absent in many hymenopteran 28S rDNA sequences (Gillespie *et al*., 2005c), including the honey bee. However, it is probable that this region of the D7a contains a cleavage site in the 28S rRNA, given that the loop formed by helix D7a-3 is extraordinarily variable in sequence length and base composition and contains no detectable conserved secondary structural elements across arthropods (e.g. Gillespie *et al*., 2005c) or other metazoan taxa studied (e.g. van Keulen *et al*., 1991; Zarlenga & Dame, 1992). Also, some insect D7a sequences contain repeated microsatellite motifs (Gillespie *et al*., 2005c) that are indicative of slippage events during replication of the rRNA gene array (Levinson & Gutman, 1987; Hancock & Dover, 1988), further suggesting that this portion of the 28S rRNA is non-functional and likely cleaved out of the mature rRNA molecule. This observation is consistent with site-directed mutagenesis studies on the yeast *Saccharomyces cerevisiae*, which show that the D7a variable region of the 26S LSU rRNA is dispensable (Musters *et al*., 1991). Recently, Basile-Borgia *et al*. (2005) provided evidence for the possible involvement of a highly conserved eukaryotic processing machinery responsible for cleavage of the α and β fragments in LSU rRNA, as microinjected *Sciara coprophila* (fungus fly) rDNA into *Xenopus laevis* oocytes resulted in transcription and pre28S rRNA fragmentation. Still, without a characterized processing machinery, an autocatalytic nature of the cleavage of cytoplasmic LSU rRNA cannot be ruled out.

Nearly half of domain IV of arthropod 28S rRNA is comprised of expansion segment D8 (Fig. 2C). Site-directed mutagenesis studies have implicated the D8 region with ribosome function (Sweeney *et al*., 1994), and it is likely that small nucleolar RNA E2 interacts with D8 in the human LSU rRNA (Rimoldi *et al*., 1993). Gillespie *et al*. (2005c) have characterized the structure of expansion segment D8 across aculeate Hymenoptera, and our analyses of more diverse taxa support their predicted structure. Domain V of arthropod 28S rRNA contains expansion segments D9, which is small and highly conserved in many Hymenoptera, including the honey bee, and D10, which is larger and highly variable in sequence composition but conserved in secondary structure (Gillespie *et al*., 2005c). As more sequences accumulate on genetic databases, it is likely that expansion segment D10 will become a more commonly used marker for phylogeny estimation, especially because it can be amplified in some arthropod taxa with all of expansion segment D12 (Gillespie, unpublished data).

**Figure 4:**
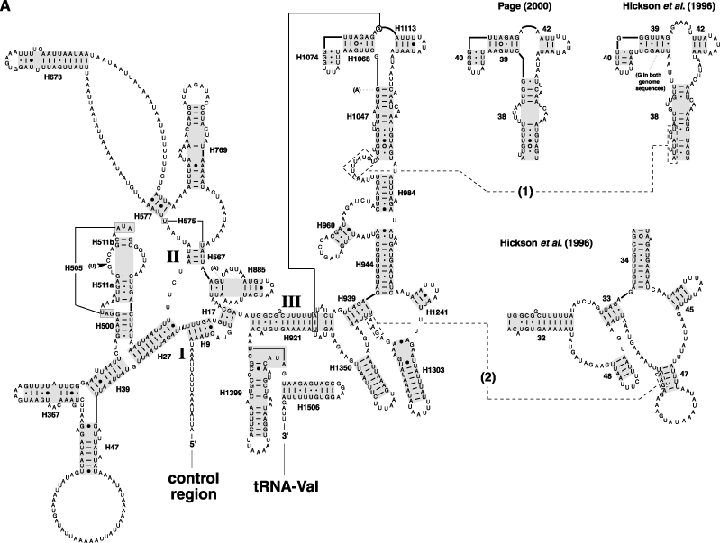

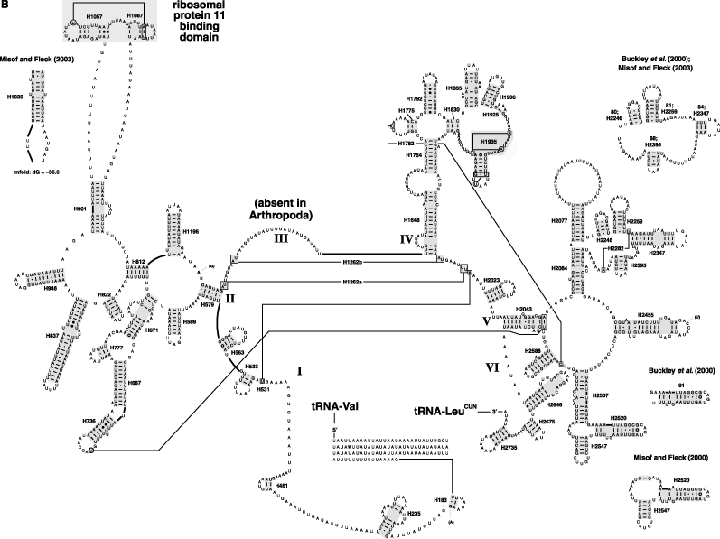
The secondary structure model of the mitochondrial rRNA (12S + 16S) from the honey bee, *Apis mellifera*. Differences between our sequence and previously published *A. mellifera* sequences (U65190 and U65191) are in bold, with insertions (dark arrows), deletions (open arrows) and substitutions (parentheses) shown. Helices aligned across all sampled panarthropods are boxed in grey. Tertiary interactions (where there is strong comparative support) and base triples are shown connected by continuous lines. (A) SSU rRNA (12S). Misaligned sequences 1 and 2 (discussed in text) are within dashed boxes and connected to redrawn structures from Hickson *et al*. (1996) and Page (2000) with dashed lines. (B) LSU rRNA (16S). See Fig. 2 legend for explanations of base pair symbols, helix numbering and reference for software used to construct structure diagrams.

Expansion segment D11 is positioned between domains V and VI in cytoplasmic LSU rRNA (Fig. 4C) and is typically short and without base pairing (Schnare *et al*., 1996). The remaining variable regions of arthropod 28S rRNA are expansion segments D12 and R2832, for which little information on base pairing can be derived given the paucity of published arthropod sequences spanning these regions (Cannone *et al*., 2002). We found minimal compensatory base change support for the four helices within expansion segment D12, and the remainder of the structured bases within this region should be considered preliminary at best. Similarly, despite having a complete 28S rRNA sequence for the honey bee, our prediction of variable region R2832 is supported minimally because most ‘complete’ published arthropod rDNA sequences are nearly complete, having the 3′-primer designed from the *D. melanogaster* sequence (it is no coincidence that the majority of published arthropod sequences containing complete or nearly complete R2832 sequences are flies). The difficulty in sequencing the entire 3′-half of the 28S rDNA is a consequence of the extreme variability within the IGS region that immediately flanks the last nucleotides of the 28S rDNA (see below).

The nuclear-encoded cytoplasmic large subunit of the ribosome contains a 5S rRNA in Archaea, Bacteria and Eukaryota, and while chloroplast and plant mitochondria also have a 5S rRNA in their large ribosomal subunit, the mitochondria in fungi, animals and many protists do not (Bullerwell *et al*., 2003). Our predicted structure of the nuclear-encoded honey bee 5S rRNA (Fig. 2D) is similar to other published structures of arthropod 5S rRNA (e.g. Barciszewska *et al*., 1995; Paques *et al*., 1995), and we confirmed our model with a recent interpretation (Szymanski *et al*., 2002) of the crystal structure of the LSU rRNA of the halophile archaea *Haloarcula marismortui* (Ban *et al*., 2000). We have no supporting evidence that this 5S rRNA, or an additional 5S rRNA encoded in the nucleus, is imported into mitochondria; however, evidence suggests that nuclear-encoded 5S rRNA is a constituent of mt ribosomes in several eukaryotes, including trypanostomatids (Mahapatra *et al*., 1994; Tan *et al*., 2002), yeast (Entelis *et al*., 2002) and mammals (Yoshionari *et al*., 1994; Magalhães *et al*., 1998; Entelis *et al*., 2001).

#### Mitochondrial SSU rRNA

In most insects, the 12S rRNA gene is flanked on the 5′-side by the non-coding portion of the mitochondrial genome that contains the control region and on the 3′-side by the valine transfer RNA (tRNA-Val). The honey bee 12S rRNA gene follows this conserved arrangement of the rRNA genes, despite seven gene rearrangements within the mitochondrial genome (Crozier & Crozier, 1993). Our structural model (Fig. 4A) follows the *Drosophila virilis* model of Cannone *et al.* (2002) with minor modifications made based on an alignment of 83 neopteran 12S rRNA sequences. Our numbering system follows the convention established at the CRW Site (see Experimental procedures). There are 30 helices within insect 12S rRNA that are supported by compensatory base change evidence across our alignment.

Other than *Drosophila* spp. (Clary & Wolstenholme, 1985, 1987), the first structural models for insect 12S rRNA were based on comparisons with other mt SSU rRNA models, such as mouse (Bibb *et al*., 1981), frog (Dunon-Bluteau & Brun, 1986), primate (Dunon-Bluteau & Brun, 1986; Hixson & Brown, 1986) and sea anemone (Pont-Kingdon *et al*., 1994). Initially used for large-scale phylogenetic analyses (e.g. Pace *et al*., 1986; Field *et al*., 1988), it soon after became clear that the phylogenetic utility of SSU mt rRNA genes was limited to lower level divergences (Thomas *et al*., 1989; Simon *et al*., 1990). Thus, a plethora of lower level studies within Insecta utilizing 12S rDNA sequences emerged. Some sequences were collected in various structural compilations (Dams *et al*., 1988; Neefs *et al*., 1993; Gutell, 1994), and together with more in-depth models on birds (Cooper, 1994), vertebrate classes (Hickson, 1993), rodents (Sullivan *et al*., 1995) and insects (Simon *et al*., 1990, 1994; Simon, 1991), Hickson *et al*. (1996) presented a refined model of animal 12S rRNA that differed from previous models in that several variable regions were adjusted from the global SSU rRNA model to fit a custom animal model [results also reached by Kjer (1995, 1997) in his analysis of amphibians]. Of particular note, the honey bee 12S rRNA sequence confounded the alignment of helix **H1047** [helix 38 of Hickson *et al*. (1996)] due to its high AT bias. This resulted in the proposal for a radical structure for this helix in honey bee. Analysing the secondary structure of 225 insect 12S rRNA sequences using maximum weighted matching, Page (2000) predicted a structure for domain III of honey bee 12S rRNA that was in more agreement with earlier insect models (e.g. Gutell, 1994). Our predicted model for domain III of honey bee 12S rRNA is more consistent with the model of Page (2000) than that of Hickson *et al*. (1996), and we have identified two regions within the alignment of Hickson *et al*. (1996) that were misaligned, causing the majority of domain III to be predicted inaccurately (Fig. 4A, boxed sequences 1 and 2). The second misaligned sequence (5′-GAA-3′) was noted by Page (2000) as being misaligned, prompting a follow-up note about the dangers of relying on conserved motifs to anchor alignments (Page, 2001). Our realignment of the first sequence (5′-AUUUAUGU-3′) further suggests that caution should be used when anchoring variable regions with thermodynamic evidence rather than support from crystal structures, as helix **H1047** contains several non-canonical base pairs in its proximal region across many arthropod taxa (Cannone *et al*., 2002). As with Page (2001), we note that, despite its misalignment in Hickson *et al*. (1996), the honey bee 12S rDNA sequence likely did not affect the analysis by Hickson *et al*. (2000), wherein their structural alignment was used to evaluate the performance of a suite of automated alignment programs.

Despite the continued use of 12S rDNA sequences, few recent studies have presented structural predictions. The predicted structures for lice (Phthiraptera) (Page *et al*., 2002), *Trirhabda* leaf beetles (Coleoptera: Chrysomelidae) (Swigonova & Kjer, 2004), species in the taxon Arthropleona (Collembola) (Carapelli *et al*., 2004) and burnet moths (Lepidoptera) (Niehuis *et al*., 2006) all slightly differ from our model in that they are refined for the lower taxonomic levels analysed in each study. This latter study is interesting because, while nearly all previous studies of 12S rRNA structure and evolution have focused primarily on domain III, Niehuis *et al*. (2006) predict a structure of the entire 12S rRNA molecule, providing primers to amplify the less common sequence domains I and II. Hopefully more studies will utilize this information, as well as our 12S rRNA structural diagram and related alignments, to compile more sequences of domains I and II from diverse taxa to improve our knowledge of this seldom-sequenced region of arthropod 12S rRNA (Cannone *et al*., 2002).

#### Mitochondrial LSU rRNA

In most insects, the 16S rRNA gene is flanked on the 5′-side by the tRNA-Val gene, and on the 3′-side by the tRNA-Leu^CUN^ gene. Like the 12S rRNA gene, the honey bee 16S rRNA gene follows this conserved arrangement (Fig. 1). Our secondary structure model (Fig. 4B) follows the *D. melanogaster* model of Cannone *et al*. (2002) with minor modifications made based on an alignment of 244 neopteran 16S rRNA sequences. Our numbering system follows the convention established at the CRW Site (see Experimental procedures). There are 49 helices within insect 16S rRNA that are supported by compensatory base changes across our alignment. Despite the presence of a complete sequence for nearly 20 years (Vlasak *et al*., 1987), we believe that this is the first published secondary structural model for honey bee mitochondrial 16S rRNA.

Since the earliest structural predictions for arthropod 16S rRNA, namely those from *Drosophila yakuba* (Clary & Wolstenholme, 1985), *Locusta* spp. (Uhlenbusch *et al*., 1987), *D. melanogaster* (Gutell & Fox, 1988) and gypsy moth, *Lymantria dispar* (Davis *et al*., 1994), more refined models have been proposed based on larger comparisons across divergent taxa, e.g. *Deltocephalus*-like leafhoppers (Fang *et al*., 1993), families of Hymenoptera (Dowton & Austin, 1994), families of Dermaptera (Kambhampati *et al*., 1996), families of Orthoptera (Flook & Rowell, 1997) and groups of spiders (Smith & Bond, 2003). These studies have undoubtedly prompted refinements to older structural diagrams present on the CRW Site and other rRNA secondary structure databases.

Buckley *et al*. (2000) analysed 16S rRNA secondary structure from over 400 taxa representing 13 insect orders. We assume that honey bee was included in their study; however, no secondary structure diagram was included for honey bee, and no honey bee sequence was present in their condensed alignment (Buckley *et al*., 2000). Aside from minor deviances, the authors noted several major differences between their predicted insect model and previous structures proposed by Gutell (unpublished then, now compiled on the CRW Site) and De Rijk *et al*. (1997). Specifically, helices 84 and 91 (our **H2347** and **H2520**) were proposed to form different base pairs within their models than in the older models, and these structures are drawn as alternatives in our 16S rRNA structural diagram (Fig. 4B). Our search across 244 neopteran 16S rDNA sequences did not yield a high amount of compensatory substitutions supporting these alternative structures (data not shown).

Consistent with most structural and phylogenetic studies on 12S rRNA (see above), nearly all studies on 16S rRNA have analysed only a portion of the 3′-half of the molecule, representing conserved domains IV and V (Fig. 4B). Recently, however, Misof & Fleck (2003) analysed domains I, II, IV and V of 16S rRNA across major groups of dragonflies and damselflies (Odonata), predicting a nearly complete model for this large insect group (domain III is absent in arthropods and domain VI is rarely sequenced due to the position of the universal 3′-primer). Much of the odonate model is consistent with our predicted 16S rRNA structure presented here, with the major differences resulting from additional base pairs proposed in regions left unpaired in our model, for example the region in domain II that interacts with ribosomal protein 11 (Fig. 4B; discussed below), and helices H1–H3 in domain I proposed by Misof & Fleck (2003). Additionally, as with Buckley *et al*. (2000), helices **H2347** and **H2520** of our model are different in the Misof & Fleck (2003) model. Interestingly, many of the regions in the odonate model that differ from our model, likely by being more odonate-based, do not reflect phylogenetic information consistent with expected relationships within Odonata (Misof & Fleck, 2003; see Hypsa, 2006).

#### Mitochondrial rRNA structural conservation.

Arthropod mt rRNA has a characteristic structure (Fig. 4) that may be correlated with genome reduction in mitochondria (e.g. Lang *et al*., 1997) such that many features common to rRNA from all domains of life are absent in the organellar rRNA structure. A lack of recombination in the mitochondria (e.g. Lynch, 1995) likely contributes to a high level of non-canonical base pairs or unstructured regions that are highly conserved in non-organellar rRNAs of other organisms. The highly oxidative nature of the mitochondria is probably responsible for mutational loads not seen in most regions of the nucleus (e.g. Martin & Palumbi, 1993), particularly in nuclear coding sequences. Rates of evolution within the mitochondrial genome are typically much faster than genes within the nucleus (e.g. Crozier *et al*., 1989; Dowton & Austin, 1995; Sullivan *et al*., 1995; Lynch, 1997; Page *et al*., 1998; Lin & Danforth, 2004), a possible consequence of the lack of recombination and proofreading, and high oxidative environment. Studies showing gene rearrangement in arthropod mtDNA are growing (e.g. Boore *et al*., 1995; Black & Roehrdanz, 1998; Campbell & Barker, 1998, 1999; Dowton & Austin, 1999; Masta, 2000; Dowton & Campbell, 2001; Shao *et al*., 2001a,b; Shao & Barker, 2003; Masta & Boore, 2004; Covacin *et al*., 2006), and it has recently been demonstrated that mt genes in arthropods show a positive correlation between high rates of nucleotide substitution and gene rearrangement (Shao *et al*., 2003). While once considered stable in their position within the arthropod mitochondrial genome, evidence for rearrangements of the rRNA genes is growing (e.g. Masta, 2000; Shao & Barker, 2003; Covacin *et al.*, 2006). It is interesting that, despite some of the highest levels of rates of substitution and AT bias (Crozier *et al*., 1989; Crozier & Crozier, 1993), the honey bee mt rRNA genes are arranged in the conserved ancestral manner of most other arthropods (Fig. 1B).

The paucity of crystal structures of mt rRNAs, coupled with the difficulty of aligning their variable regions due to a high AT bias, does not permit us to evaluate accurately the regions of mt rRNAs that are truncated or deleted relative to the cytoplasmic rRNAs. Ideally, significant differences from our conserved structural model, i.e. the louse 12S rRNA model (Page *et al*., 2002) or the odonate 16S rRNA model (Misof & Fleck, 2003), should be evaluated across all published arthropod mt rRNA sequences to determine the base pairings with the strongest positional covariation. Unfortunately, this difficult task is beyond the scope of our current investigation. However, we stress that regions of insect mt rRNA that differ across diverse taxa in both base composition and predicted secondary structures, may in fact be conserved in tertiary interactions within the ribosome. For example, the ribosomal protein 11 binding-domain within domain II of LSU rRNA (Fig. 4B) and associated ribosomal protein 11 (L11) is one of three structural domains that are proximal on the 50S subunit, forming the GTPase-associated centre (Wimberly *et al*., 1999). Recent evidence suggests that the structure of the LSU rRNA stabilized by the C-terminal domain of L11 is necessary to alleviate elongation factor G (EF-G) binding in the post-translocation state of the ribosome (Bowen *et al*., 2005). In arthropod 16S rRNA sequences between the L11 binding-domain and helix **H991**, which are shorter and closer to the core rRNA due to the loss of pseudoknot **H1005** and helices **H1011** and **H1030**, nucleotides must remain unpaired and in a ‘stretched’ conformation in order for the L11–L11 binding domain to proximally interact with two other structural domains in the GTPase-associated centre of the LSU rRNA. Thus, base pairing in these regions, even if supported by compensatory base change evidence (which by chance alone would outperform unpaired sites; see mfold structure in Fig. 4B), would be deleterious to ribosome assembly and function, as recently suggested for mammalian 16S rRNA (Mears *et al*., 2006). Given this, we doubt the formation of secondary structure in this region of 16S rRNA, although, as in the case of V4 of 18S rRNA (Fig. 3), it cannot be ruled out that base pairing in these regions may be temporary, ultimately dissolving during the tertiary interactions formed before and during translation. However, unlike the V4 region of 18S rRNA, transitionally less optimal structures are so far not supported across all arthropod sequences, and hence cannot support a hypothetical dynamic nature of this region of LSU rRNA.

Taxon-specific structures in mt rRNA variable regions would seemingly imply changes to associated ribosomal proteins and other ribosome cofactors, such that all of these molecules would coevolve to maintain the structural integrity and function of the mt ribosome. This is unlikely as ribosomal proteins are usually highly conserved across deep levels of divergence (e.g. Graybeal, 1994; Koonin *et al*., 2004; Stuart & Berry, 2004). Studies characterizing the coevolution of mitochondrial ribosomal proteins and their variable rRNA counterparts are needed to determine, if any, the secondary structures that are homologous across insects.

### Characterization of IGS- and ETS-rDNA boundaries

Despite the growing number of sequenced eukaryotic genomes, few studies have predicted full sequences spanning the entire IGS, especially in arthropods. Usually, only those studies that sequence clones report complete IGS sequences, as intergenomic variation compounds other simpler methods. For genome sequencing studies, two main problems confound the accurate assemblage of IGS sequences: variation in subrepeat number, and the presence of multiple subrepeats that are not contiguous (i.e. separated by conserved or other repetitive sequences). This second problem is critical because if the variation within subrepeats is high then multiple non-contiguous subrepeats can be spuriously assembled in the wrong locale of the IGS, ultimately compounding the rebuilding of the accurate IGS sequence. Methods for accommodating these problems are in great need, as IGS regions contain the rDNA promoter region, as well as enhancer sequences that facilitate transcription of arthropod rRNA genes (e.g. Hayward & Glover, 1988). These regions are also useful for species identification in arthropods (e.g. McLain *et al*., 1989; Pepera *et al*., 1998; Mukha *et al*., 2000; Whang *et al*., 2002; Ohnishi & Yamamoto, 2004) and assessing the fitness and the evolutionary ecology of organisms (e.g. Cluster *et al*., 1987; Weider *et al*., 2005).

Relative to coding regions of the rRNA genes, complete IGS sequences from arthropods have only been characterized for a few taxa, and many of these are dipterans: *D. melanogaster* (Simeone *et al*., 1985; Tautz *et al*., 1987; Hayward & Glover, 1988; Tautz *et al*., 1988), *D. orena*, *D. virilis*, *D. hydei* (Tautz *et al*., 1987), *Aedes albopictus* (Baldridge & Fallon, 1992), *A. aegypti* (Wu & Fallon, 1998), *Anopheles sinensis* (Whang *et al*., 2002), the tstetse fly, *Glossina* sp. (Glossinidae) (Cross & Dover, 1987a,b), the black fly, *Simulium sanctipauli* (Simuliidae) (Morales-Hojas *et al*., 2002) and several species of *Chironomus* midges (Chironomidae) (Degelmann *et al*., 1979; Israelewski & Schmidt, 1982; Israelewski, 1983). Other published arthropod complete IGS sequences are from the German cockroach *Blattella germanica* (Blattaria: Blattellidae) (Mukha *et al*., 2002), the bulldog ant, *Myrmecia croslandi* (Hymenoptera: Formicidae) (Ohnishi & Yamamoto, 2004) and the crustaceans *Daphnia pulex* (Cladocera) (Crease, 1993) and *Tigriopus californicus* (Copepoda) (Burton *et al*., 2005). Much of the IGS and ETS have also been characterized for the pea aphid, *Acyrthosiphon pisum* (Kwon & Ishikawa, 1992) and the silkmoth, *Bombyx mori* (Fujiwara & Ishikawa, 1987). In agreement with complete IGS sequences from other eukaryotes, including the nematode *Caenorhabditis elegans* (Ellis *et al*., 1986), several diverse plants (Gruendler *et al*., 1991; Lanadu *et al*., 1992; Lakshmikumaran & Negi, 1994; Rocheford, 1994; Chiang *et al*., 1998; Fernández *et al*., 2000; Macas *et al*., 2003; Hsieh *et al*., 2004) and trypanostomatids (Schnare *et al*., 2000; Orlando *et al*., 2002; Boucher *et al*., 2004), IGS sequences contain one to several ‘types’ of repeat regions that are variable within species and difficult to compare across even closely related species (e.g. Murtif & Rae, 1985). Because these repetitive regions are associated with the rDNA transcription promoter, it is no surprise that RNA polymerase I complexes of one species fail to transcribe the rRNA genes of another (e.g. Dover & Flavell, 1984; Dover & Tautz, 1986).

#### IGS

**Figure 5:**
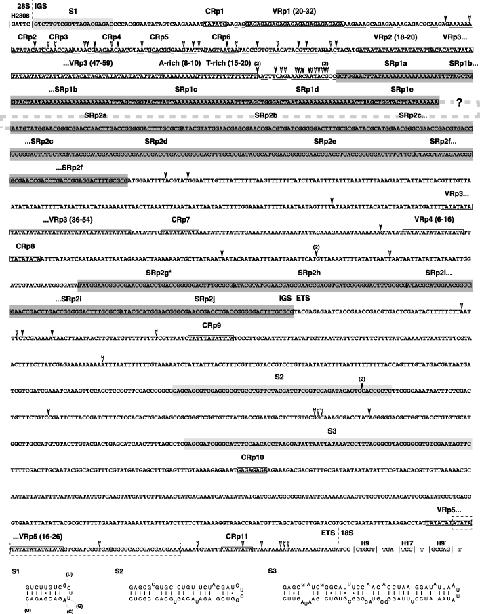
Regions of the IGS and ETS flanking the 3′-half of the 28S rDNA and the 5′-half of the 18S rDNA in the honey bee. Diagram depicts a consensus of assembled contigs, with infrequent insertions (filled arrows) and deletions (open arrows) shown in conserved regions of the alignment, as well as in conserved repetitive (CRp) regions. Length variation within variable repetitive (VRp) regions is given as ranges. Variation in sequence length and base composition is not provided for sub-repetitive (SRp) regions. All repetitive regions, as well as single-base length variable regions, are boxed and contiguous (SRp) regions are darkly shaded. The region of the IGS for which sequence identity was not possible due to intragenomic heterogeniety is depicted with a dashed line linking two large (SRp) regions. A putative promoter sequence, as predicted with the Neural Network Promoter Prediction tool (Reese, 2001) at the Berkeley Drosophila Genome project (http://www.fruitfly.org/seq_tools/promoter.html) is within a dashed box, with the bolded nucleotide depicting the predicted transcription start site. Conserved rRNA helices flanking the IGS (**H2808**) and ETS (**H9**) are within vertical bars (|). Regions with putative secondary structures (**S1–S3**) are lightly shaded, with structural diagrams shown below the alignment. The structure for S1 is shown with variable bases in a related hymenopteran, *Myrmecia croslandi*, shown in parentheses. Alignments are available at the jRNA website.

We predicted the sequence and structure of the conserved regions flanking the 3′-end of the 28S rDNA (473 nts of the IGS) and the 5′-end of the 18S rDNA (2042 nts of the IGS + ETS) mostly from the repeat reads of assembly 3 (Fig. 5). While a distinct boundary between the IGS and ETS was not determined, we treat the description of the ETS separately (next section). Immediately flanking the boundary of the 28S rDNA and the IGS we predict a helix of eight base pairs that can also form in the bulldog ant (**S1** in Fig. 5). Interestingly, Ohnishi & Yamamoto (2004) predicted a compound helical region in this same position in bulldog ant consisting of over 639 nts. While not experimentally proven, it is possible that this region of the IGS fosters a helical element; however, structural prediction in honey bee yielded nothing substantially significant other than helix **S1** (data not shown). The remaining nts of the IGS flanking the 3′-end of the 28S rDNA contain six conserved repetitive (CRp) regions, three variable repetitive (VRp) regions, one A-rich region, one T-rich region and a subrepetitive (SRp) region (Fig. 5). This first SRp region, SRp1, is estimated to repeat from four to 11 times in the honey bee genome, but could likely exceed this range. Due to the variability in sequence length and base composition of SRp1, we could not accurately predict sequences flanking the 3′-end (dashed line in Fig. 5).

As with the region of the IGS flanking the 3′-end of the 28S rDNA, the region of the IGS adjacent to the ETS cannot be predicted beyond a SRp region (Fig. 5). This second SRp region, SRp2, is more complicated than SRp1 because it is repeated at least twice within conserved stretches of sequence. SRp2 is present from three to six times (and likely more), and is then interrupted by a conserved sequence containing two CRp regions and two VRp regions (Fig. 5). SRp2 then repeats again from two to four times before flanking a conserved stretch of 1113 nts that is likely comprised mostly of the ETS. It is difficult to predict the composition and structure of the internal region of the IGS flanked by SRp1 and SRp2 for two reasons. First, the number of SRp regions vary in arthropods, ranging from one in the pea aphid (Kwon & Ishikawa, 1992) to three in *D. melanogaster* (Tautz *et al*., 1988). Thus, although we identified two distinct SRp regions in honey bee IGS, a third (or more) SRp region may exist in the central portion of the spacer. It is interesting, however, that the bulldog ant has two SRp regions, and this may be a characteristic of hymenopteran IGS sequences. Second, the composition of the central portion of the IGS in arthropods is variable, in that it can be comprised entirely of repetitive regions (e.g. bulldog ant) or alternating conserved and repetitive regions (e.g. *A. sinensis*). While we have not reported a complete sequence for the IGS from honey bee, we predict that our characterization of the conserved sequences flanking two SRp regions will allow for primer design and sequencing of the remaining internal sequence.

#### ETS

Three CRp regions and one VRp region occur within a highly conserved stretch of nts that contains the rDNA gene promoter and probable transcription enhancer elements. Spacer and gene promoters have been determined for several arthropods (listed in Crease, 1993), with a consensus sequence of 5′-TA-TATANGRRRR-3′ accounting for the variation across diverse taxa. We did not find a sequence within the putative ETS region of honey bee that matched this motif; however, using the Neural Network Promoter Prediction tool (Reese, 2001) at the Berkeley Drosophila Genome project (http://www.fruitfly.org/seq_tools/promoter.html), a promoter sequence 5′-ATATATATATATATATATAGTCGATCGGTGAGGGGGCACC**G**ACGACGAAA-3′ was predicted with a score of 1.0 (Fig. 5). A second promoter sequence 5′-ATTATATATATATATATATATATATATAGTCGATCGGT**G**AGGGGGCACCG-3′, shifted nine nts upstream from the first predicted promoter, was predicted with a score of 0.93. The bold nucleotide in each sequence is the predicted transcription initiation site (TIS), and the predicted TIS in the more optimal promoter is shaded in Fig. 5. Despite high scores, these promoter sequences are likely too close to the start of the 18S rDNA, especially when compared to the location of promoters in other arthropod ETS sequences. These putative promoter sequences and TIS should be taken with caution, as it will be necessary to determine the true promoter and TIS with S1 nuclease mapping.

Despite the high level of variation across ETS regions from different taxa, some characteristics are conserved and may allude to functional constraints not easily identifiable from primary sequence comparison. For instance, the GC content within the putative ETS region of honey bee is 35.3% and similar to other holometabolous insects; e.g. *B. mori* (36.7%) (Fujiwara & Ishikawa, 1987), *D. melanogaster* (24%) and *D. orena* (23.7%) (Tautz *et al*., 1987) and tsetse fly, *Glossina morsitans* (28%) (Cross & Dover, 1987a). The GC content of a non-holometabolous insect, the pea aphid, is 69% (Kwon & Ishikawa, 1992), indicating a possible correlation between base composition in the ETS and phylogeny. Helical elements have been predicted within the ETS region in other eukaryotes (Fernández *et al*., 2000; Schnare *et al*., 2000; Orlando *et al*., 2002; Boucher *et al*., 2004) and likely have an important role in the regulation of transcription and processing of rRNA. Using secondary structure, these regions can be aligned across closely related species (Fernández *et al*., 2000; Schnare *et al*., 2000; Orlando *et al*., 2002), providing information on putative rRNA processing sites (Schnare *et al*., 2000). Within the honey bee ETS region we calculated two putative helices using mfold (Mathews *et al*., 1999; Zuker, 2003) that warrant further validation from closely related hymenopteran taxa (Fig. 5). We were unable to identify these helices in the bulldog ant; however, given the variability present in other ETS helical elements, e.g. in legumes (Fernández *et al*., 2000) or trypanostomatids (Schnare *et al*., 2000), the bulldog ant and honey bee may have very divergent structures involved in the processing of the ETS region.

### Retrotransposition in 28S rDNA sequences

#### R2-element insertion sites

**Figure 6:**
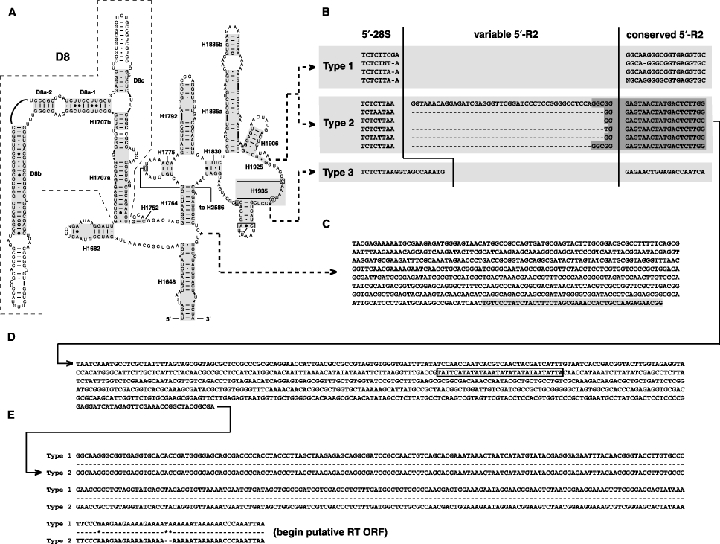
R1 and R2 element insertion sites in honey bee 28S rDNA sequences, and variable R2 element 5′-UTRs. (A) Predicted secondary structure of domain IV of honey bee 28S rRNA, with asterisks depicting R1 and R2 element insertion sites. (B) Variable 5′-junction of R2 element insertion sites. Shaded region in type II elements depict conserved regions flanking a highly variable junction. (C) Three-prime junction of partial putative R1 element in honey bee 28S rDNA. Shaded sequence depicts 28S rDNA. Note: no 5′-junction was recovered for this partial R1 element (see text). (D) Conserved 758 nts in the 5′-UTR of type II R2 elements. Boxed sequence contains variation across unassembled reads. (E) Conserved 335 nts of the 5′-UTR of type I and type II R2 elements. See  Fig. 2 legend for explanations of base pair symbols, helix numbering and reference for software used to construct structure diagrams.

**Figure 7:**
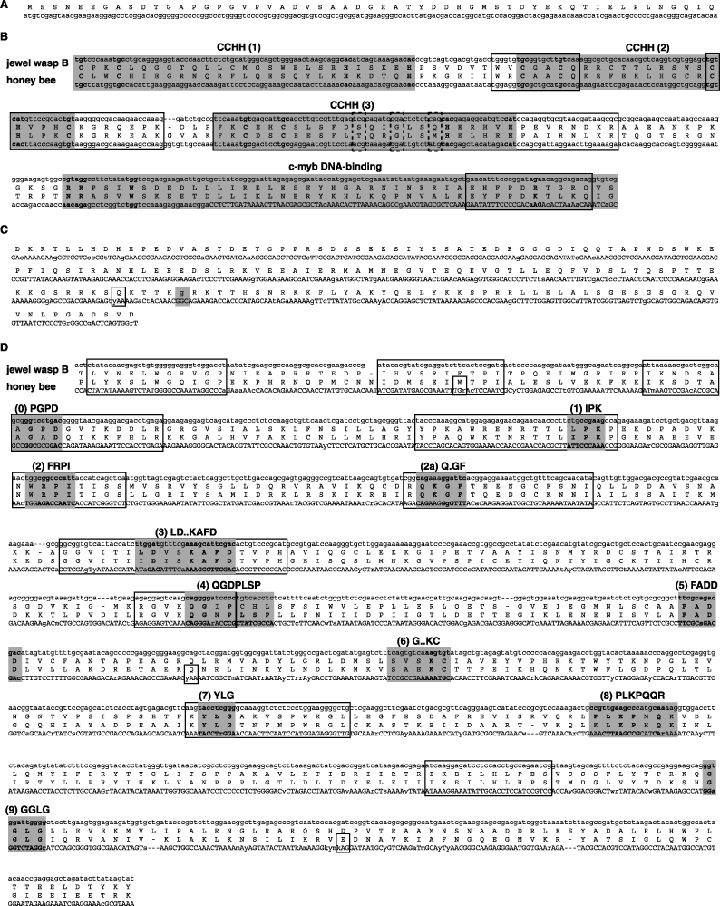

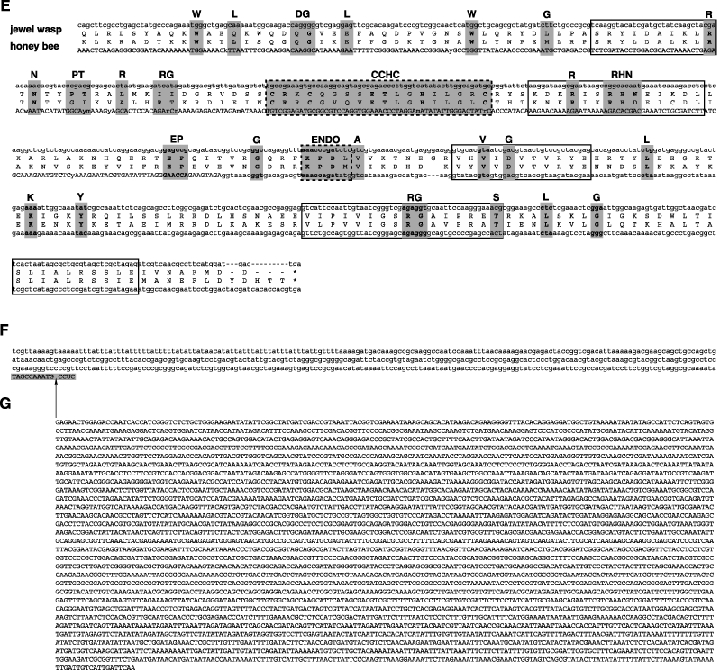
Characteristics of the honey bee R2 element ORF, 3′-UTR and potential imprecise insertion site. Bold amino acid residues are conserved across 80% or more arthropod R2 elements (Burke *et al*., 1999). Boxed aligned regions depict highly conserved sequence across parasitic Hymenoptera. Single-boxed residues depict codons with IUPAC ambiguity codes with the alternative nucleotide causing a stop codon. (A) Non-conserved 5′ sequence of the ORF with the putative initiation codon *Met*. (B) Comparison of jewel wasp B sequence (*Nasonia* sp., Pteromalidae) (GenBank accession no. AF090145) to honey bee in the conserved amino-terminal domains. Shaded regions depict conserved motifs of DNA-binding proteins, with dashed boxes showing the three residues believed to interact with the α-helical region of DNA. CCHH, Cys-His motifs; c-myb, proto-oncogene protein. (C) Non-conserved sequence flanked by the conserved amino-terminal motifs and the 5′-end of the RT domain. Note: the jewel wasp B sequence contains 255 amino acid residues compared to the 178 of the honey bee. The shaded glycine residue was recovered in only one unassembled read. (D) Comparison of jewel wasp sp. B and honey bee in the highly conserved reverse transcriptase (RT) domain, including the fingers/palm and thumb motifs. The 11 shaded regions depict motifs conserved in the RTs of all retroelements (Xiong & Eickbush, 1990; Burke *et al*., 1999). (E) Comparison of jewel wasp B and honey bee in the conserved carboxyl-terminal domains. The DNA-binding motif CCHC and the KPDI sequence (ENDO) are shaded and within dashed boxes, representing the endonculease domain. Other shaded motifs depict conserved residues in arthropods (Burke *et al*., 1999). (F) Predicted 3′-UTR. The shaded sequence is the 3′-junction with the 28S rDNA (see Fig. 5), with the space depicting the potential imprecise insertion site of R2 element type 3. (G) Consensus sequence of R2 element type 3, which seemingly inserts 12 nucleotides downstream from the typical R2 insertion site. No ORF has been predicted for this element (see text).

We report the presence of three distinct R2 elements inserted into the 28S rDNA at the typical target sites described for other arthropods (e.g. Long & Dawid, 1979; Jamrich & Miller, 1984; Jakubczak *et al*., 1990, 1991; Burke *et al*., 1993, 1999; Eickbush, 2002). In general, most arthropod R2 elements insert between nucleotides 1928 and 1929 of the lonepair triloop (LPTR) **H1925**, however, R2 elements have also been shown to insert in helices **H1835a** and **H1906** (Jakubczak *et al*., 1991). It now appears that only the initial nick in the target 28S rDNA sequence is conserved across arthropods, with the cleavage of the second strand highly variable such that the 5′-junctions of the R2 element and 28S rDNA are highly variable in sequence length and base composition, even within species (Burke *et al*., 1999). Indeed, our findings suggest at least two types of R2 element insert within nucleotides 1928 and 1929 of LPTR **H1925** (Fig. 6A, B). Type 1 R2 elements are more conserved at their 5′-junction with the 28S rDNA and have a putative 5′-untranslated region (5′-UTR) of approximately 335 nts (Fig. 6E). In contrast, type 2 R2 elements are extraordinarily variable in their junction with the 28S rDNA and have a putative 5′-UTR of approximately 1039 nts (Fig. 6D,E). The 3′-region of this larger 5′-UTR from type 2 R2 elements is nearly identical with the entire 5′-UTR of type 1 R2 elements (Fig. 6E), suggesting that the additional nucleotides in the 5′-half of the 5′-UTR of type 2 R2 elements are not likely contributing to the structure and function of the conserved 5′-UTR. The ultimate 3′-nucleotides of the 5′-UTR in all type 1 and type 2 R2 elements from honey bee flank a predicted start codon of the putative ORF of the RT protein (Figs 6E and 7A).

We also discovered a third type of R2 element in honey bee 28S rDNA. Type 3 R2 elements were found to insert in an unusual position in the 28S rDNA, helix **H1935** (Fig. 6A,B), and did not reveal a typical ORF of other arthropod R2 elements (Fig. 6G). This, coupled with a lack of a 3′-junction with the 28S rDNA, and the fact that we only detected a few copies of type 3 R2 elements, led us to doubt that this position in honey bee 28S rDNA fosters the insertion of R elements. It could also be that this particular region of 28S rDNA was erroneously assembled in the building of the genome. Still, as the number of arthropod genome sequences continue to grow, it is likely that new insertion sites in 28S rDNA will be discovered, especially within this region of domain IV, given that it is one of the most interrupted regions of rDNA across all domains of life (Jackson *et al*., 2002).

#### Predicted R2-element ORF and 3′-UTR

Given that ORFs for the RT of R2 elements have been predicted for a variety of arthropods (e.g. Burke *et al*., 1999; Kojima & Fujiwara, 2004, 2005; Bunikis & Barbour, 2005), we aligned and predicted the domain structures for honey bee R2-RT sequences by comparison with another hymenopteran insect, the jewel wasp *Nasonia* sp. (Chalcidoidea: Pteromalidae) (Fig. 7B,D,E). *Nasonia* sp. is remarkable because it contains two different types of R2 elements: one that is comparable to most other arthropod R2 elements (type A), and a second that differs mainly in the presence of two additional zinc-finger domains in the amino-terminal region (type B) (Burke *et al*., 1999). Only one other arthropod, the horseshoe crab *Limulus polyphemus*, has a type B R2 element (Burke *et al*., 1999). Interestingly, not only does our analysis of the honey bee genome identify the presence of a type B R2 element similar to horseshoe crab and *Nasonia* sp. type B elements (a finding consistent with Kojima & Fujiwara, 2005) (Fig. 7B), it also suggests that the honey bee does not have the more common type A R2 element. Within a phylogenetic context of Hymenoptera (e.g. Dowton & Austin, 1994), wherein Chalcidoidea is derived in regards to a more primitive Apidae, this implies that *Nasonia* sp. has retained both A and B type R2 elements, whereas honey bee has lost the more typical A type but retained the less frequent B type, which is likely a more primitive parasite of arthropod genomes (Kojima & Fujiwara, 2005). Two types of R2 elements have been detected in the cicada killer, *Sphecius speciosus* (Sphecoidea; Sphecidae); however, the limited portion of the published sequence cannot lead us to determine if this insect contains type A or B R2 elements (or both). In response to this, we have identified several regions within the ORF alignment of *Nasonia* sp. B and honey bee R2 elements that are highly conserved for the design of PCR and sequencing primers (Fig. 7). We are currently using primers from these regions to determine the presence or absence of type B R2 elements across hymenopteran families. Given that R2 elements typically evolve at rates similar to their rDNA hosts (Eickbush & Eickbush, 1995; Eickbush *et al*., 1995), we expect our investigation to yield a reasonable estimate of the evolution of R2 elements across Hymenoptera, and the degree of vertical and horizontal transmission of these mobile elements.

The remaining conserved domains of the honey bee R2 element ORF, namely the RT domain with fingers/palm and thumb motifs (Fig. 7D) and the carboxyl-terminal domains that include the CCHC DNA-binding motif and endonuclease domain (Fig. 7E), are highly conserved across honey bee and jewel wasp. The sequence between the ORF stop codon (UGA) and the 3′-junction of the 28S rDNA, or 3′-UTR, is more conserved than the 5′-UTR and comprises 504 nts. Thus, it is similar in size to the *Nasonia* spp. 3′-UTR, which is 594 nts (Burke *et al*., 1999). While predicted secondary structures exist for R2 element-3′-UTRs from several arthropods, including the lepidopterans *Bombyx mori* (silkmoth), *Samia cynthia* (*Ailanthus* silkmoth), *Callosamia promethea* (*Promethea* silkmoth), *Coscinocera hercules* (Hercules moth) and *Saturnia pyri* (great peacock moth), the earwig (*Forficula auricularia*) and several *Drosophila* spp. (Mathews *et al*., 1997; Ruschak *et al*., 2004), no secondary structures have been predicted for hymenopteran insects. This is likely due to the large size of the hymenopteran R2 element-3′-UTR as compared to other arthropods, but also due to a lack of more sequences to facilitate the comparative method for predicting structure. Even using the *Nasonia* spp. (A and B) for comparative analysis, our attempts at predicting a secondary structure for honey bee R2 element-3′-UTR were futile.

While non-LTR retrotransposable elements are likely highly abundant in arthropod genomes (Perez-Gonzalez & Eickbush, 2001), most copies are likely defective elements that drift with their inactivated rRNA gene hosts (e.g. Jamrich & Miller, 1984; Weiner *et al*., 1986; Luan & Eickbush, 1996; Sassaman *et al*., 1997). Subject to the process of concerted evolution or biased gene conversion of rDNA arrays (Bigot *et al*., 1992; Jakubczak *et al*., 1992), non-functional R2 elements within inactivated rRNA genes are rapidly eliminated from arthropod genomes (Perez-Gonzalez & Eickbush, 2001, 2002). Despite the likely non-functional putative type 3 R2 element described above, we found limited evidence for non-functional R2 elements within the rRNA genes of the honey bee. This is probably an artefact of our method for constructing the R2 element sequences from conserved flanking regions of 28S rDNA, as the majority of non-functional R2 elements would likely reside within heavily mutated 28S rDNA sequences, which would not have been sampled in our procedure. Of the conserved R2 elements with putatively functional ORFs, we identified four nucleotide positions that contain variation in excess of single nucleotide polymorphisms (SNPs). All four cases pertain to either first or third codon positions of predicted amino acids, with the state in less frequency resulting in a stop codon (Fig. 7C,D). These variable positions likely indicate the presence of some non-functional R2 elements that have yet to be pruned out of the genome by concerted evolution of rRNA genes.

#### Lack of functional R1 elements in the honey bee genome

R1 elements are found in arthropod 28S rRNA genes in a conserved insertion site 74 nts downstream of the R2 insertion site (Roiha *et al*., 1981) (Fig. 6A). Our search for R1 elements within the honey bee genome did not recover any functional or complete sequences. Only truncated copies of putative R1 elements were assembled at the 3′-junction of the 28S rDNA (Fig. 6C). It is surprising that the honey bee does not contain complete and functional R1 elements, given that other hymenopteran insects have been purported to harbour them (Jakubczak *et al*., 1991; Bigot *et al*., 1992; Varricchio *et al*., 1994). According to Jakubczak *et al*. (1991), *Nasonia* sp., *S. speciosus and* the carpenter bee, *Xylocopa* sp., all contain R1 elements, although ORFs have not been determined for any of these species, and only the 3′-junctions with the 28S rDNA were actually sequenced. Similarly, Bigot *et al*. (1992) detected the presence of R1 elements in 12 Hymenoptera, including the honey bee; however, the distinction between functional and truncated copies could not be determined by Southern blot analysis. The results of their RFLP analysis of the R1 insertion site (Fig. 3, Bigot *et al*., 1992) showed a small amount of DNA in honey bee as compared to other sampled Hymenoptera, a finding consistent with our detection of only truncated R1 elements. Indeed, 5′-truncated R1 elements of sizes 0.5 and 1 kb long are known from the 28S rRNA genes of *D. melanogaster* (Jakubczak *et al*., 1990, 1991). In agreement, our truncated consensus R1 element is 577 nts long, and thus could reflect the remnant of once functional R1 elements in the genome of the honey bee that have since been pruned by rRNA gene homogenization. Alternatively, R1 elements could occur in such low frequency in the honey bee genome that no functional copies were included in its assembly.

## Conclusion

Our analysis of the rRNA genes from the honey bee suggests that the functional rRNA-coding regions are structurally conserved and homogeneous throughout the nuclear and mitochondrial genomes. Some regions of mt rRNAs that are variable in sequence length and base composition do not contain secondary structures that are conserved across Insecta. Studies evaluating these variable regions within the tertiary structure of the ribsome, including rRNA-rRNA and rRNA–ribosomal protein interactions, are needed to determine their structural and functional significance. Our preliminary characterization of the IGS and ETS regions linking nl rRNA genes in the honey bee suggests that these highly variable sequences are relatively similar to other holometabolous insects in organization, repetitive nature and base composition. Like most other arthropods studied, honey bee rRNA genes are subject to parasitism by retrotransposable elements, although they lack both the most common type of R2 element and functional R1 elements. In Hymenoptera, it has been hypothesized that the intriguing haplo-diploid system, in which males come from parthenogenic eggs (*n*) and females come from fertilized eggs (2*n*), is correlated with a low level of genetic variability, relative to other arthropod genomes (Grauer, 1985; Woods & Guttman, 1987; Bigot *et al*., 1992). The characteristics of the honey bee rRNA genes we present here cannot contest this claim.

## Experimental procedures

### rRNA secondary structure prediction

The assembled honey bee rDNA sequences were integrated into the arthropod rRNA models (nl 18S, 5.8S, 28S, 5S rRNAs; mt 12S and 16S rRNAs) predicted and compiled at the Comparative RNA website (CRW Site) (http://www.rna.icmb.utexas.edu) and the jRNA website (http://hymenoptera.tamu.edu/rna). Helix numbering follows the *E. coli* system available at the CRW Site. Information pertaining to the alignment of RNA sequences using secondary structure models, including covariation analysis, thermodynamic algorithms, and ambiguously aligned regions, is available at both websites. Secondary structure model diagrams were generated with the program XRNA (developed by B. Weiser and H. Noller, University of Santa Cruz, CA). Base pair frequency tables and structural diagrams are available at the CRW Site. Differences between our previous arthropod rRNA structures and those presented here are illustrated at the jRNA website.

### IGS sequence comparison

Conserved rDNA sequences flanking the 3′-terminal end of the 28S rRNA and 5′-terminal end of the 18S rRNA were used for Blast (Altschul *et al*., 1990) searches at the Honey Bee Genome Database (http://racerx00.tamu.edu/bee_resources.html). All options in assembly 3 were explored, including unassigned groups (bin 0); however, almost without exception, all Blast results were within the repeat reads of assembly 3. Default Blast settings were used, except that we did not filter for low complexity. Results were viewed as master-slave with identities and displayed with 500 descriptions and alignments. We used master-slave to identify reads used in subsequent Blast searches to extend the sequence. Only sequence differences repeated four or more times were reported. Unique and rare SNP differences were not reported. Sequences were then aligned manually in SeAl v2.0a11 (Rambaut, 1996). Only one alignment was made for the 5′-end of the IGS, while three alignments were made for the 3′-end of the IGS and ETS regions. Alignments are available at the jRNA website.

R1 and R2 element prediction rDNA sequences spanning the conserved insertion sites for R1 and R2 elements in arthropods were compiled using the Blast strategy discussed above. Results containing non-rDNA sequences inserted either at the 5′- or 3′-end of the rDNA insertion site were exported to SeAl for manual alignment. Further Blast searches were performed using conserved regions of aligned sequences, allowing us to ‘walk’ across the R elements from both the 5′- and 3′-ends. Upon completion of the R2 elements, we translated the consensus sequence in six frames to determine the ORF of RT protein. This allowed for the identification of putative start and stop codons. Nucleotide and amino acid sequences of honey bee R2 element were aligned with the R2-B element of the jewel wasp, *Nasonia* sp. (GenBank accession no. AF090145). Alignment was performed manually with reference to the published structure of RT proteins from arthropods (Burke *et al*., 1999).

## Supplementary material

The following material is available for this article online:

S1. Base pair frequency table comparing the models of Wuyts *et al*. (2000) and Ouvrard *et al*. (2000) for variable region 4 (V4) of arthropod 18S rRNA.

S2. Alignments of the IGS and ETS regions of unassembled honey bee rDNA sequences.

S3. Alignments of R1 and R2 retrotransposable elements of the honey bee.

This material is available from the Comparative RNA website, http://www.rna.icmb.utexas.edu, and the jRNA website http://hymenoptera.tamu.edu.

## Note added in proof

Carapelli *et al.* (2006) recently predicted the secondary structure of the mt 16S rRNA for the strepsipteran *Xenos vesparum*. There are significant differences between their model and the model presented here, particularly in domain V of mt LSU rRNA.

Ref:

CarapelliAVanniniLNardiFBooreJLBeaniLDallaiRFratiFThe mitochondrial genome of the entomophagous endoparasite *Xenos vesparum* (Insecta: Strepsiptera)Gene20063762482591676614010.1016/j.gene.2006.04.005
